# Next Generation Sequencing Improves the Accuracy of *KRAS* Mutation Analysis in Endoscopic Ultrasound Fine Needle Aspiration Pancreatic Lesions

**DOI:** 10.1371/journal.pone.0087651

**Published:** 2014-02-04

**Authors:** Dario de Biase, Michela Visani, Paola Baccarini, Anna Maria Polifemo, Antonella Maimone, Adele Fornelli, Adriana Giuliani, Nicola Zanini, Carlo Fabbri, Annalisa Pession, Giovanni Tallini

**Affiliations:** 1 Department of Medicine (DIMES) – Anatomic Pathology Unit, Bellaria Hospital, University of Bologna, Bologna, Italy; 2 Department of Pharmacology and Biotechnology (FaBiT), University of Bologna, Bologna, Italy; 3 Unit of Gastroenterology, Azienda Unità Sanitaria Locale di Bologna - Bellaria Hospital, Bologna, Italy; 4 School of Gastroenterology, University of Ferrara, Ferrara, Italy; 5 Anatomic Pathology Unit, Azienda Unità Sanitaria Locale di Bologna - Maggiore Hospital, Bologna, Italy; 6 Indiana University, Bloomington, Indiana, United States of America; 7 Unit of General Surgery, Azienda Unità Sanitaria Locale di Bologna - Maggiore Hospital, Bologna, Italy; UT MD Anderson Cancer Center, United States of America

## Abstract

The use of endoscopic ultrasonography has allowed for improved detection and pathologic analysis of fine needle aspirate material for pancreatic lesion diagnosis. The molecular analysis of *KRAS* has further improved the clinical sensitivity of preoperative analysis. For this reason, the use of highly analytical sensitive and specific molecular tests in the analysis of material from fine needle aspirate specimens has become of great importance. In the present study, 60 specimens from endoscopic ultrasonography fine needle aspirate were analyzed for *KRAS* exon 2 and exon 3 mutations, using three different techniques: Sanger sequencing, allele specific locked nucleic acid PCR and Next Generation sequencing (454 GS-Junior, Roche). Moreover, *KRAS* was also tested in wild-type samples, starting from DNA obtained from cytological smears after pathological evaluation. Sanger sequencing showed a clinical sensitivity for the detection of the *KRAS* mutation of 42.1%, allele specific locked nucleic acid of 52.8% and Next Generation of 73.7%. In two wild-type cases the re-sequencing starting from selected material allowed to detect a *KRAS* mutation, increasing the clinical sensitivity of next generation sequencing to 78.95%. The present study demonstrated that the performance of molecular analysis could be improved by using highly analytical sensitive techniques. The Next Generation Sequencing allowed to increase the clinical sensitivity of the test without decreasing the specificity of the analysis. Moreover we observed that it could be useful to repeat the analysis starting from selectable material, such as cytological smears to avoid false negative results.

## Introduction

Pancreatic ductal adenocarcinoma (PDAC) represents the fourth-highest cause of cancer death in the United States with the lowest survival rate among the most common cancers (∼6%) [1]. Several imaging techniques have been developed to improve early diagnosis of pancreatic masses, such as multi-detector-row computed tomography (MDCT), transcutaneous ultrasonography (TUS), magnetic resonance imaging (MRI), endoscopic ultrasonography (EUS), endoscopic retrograde cholangiopancreatography (ERCP) and positron emission tomography (PET) scanning [2]–[4]. Among these techniques, endoscopic ultrasonography guarantees the highest-resolution imaging of the pancreas, allowing for the detection of small masses [5], of lymph node involvement [2] and of vascular tumor infiltration [3]. The introduction of the EUS-guided fine needle aspiration (EUS-FNA) in the clinical practice has supported clinicians in the preoperative diagnosis of pancreatic tumors helping to correctly and promptly selecting patients eligible for a curative surgical intervention or for other treatment [4], [6], [7].

Although EUS-FNA shows high diagnostic clinical sensitivity and specificity, a subset of cases are characterized by limited cellularity or inadequate material for cytologic evaluation [8]. Other than these “unsatisfactory” specimens, inconclusive cytologic cases include those samples described as “suspicious of malignancy” or with “presence of atypical cells” which also represent a significant problem for clinicians and pathologists. The combination of cytologic evaluation and molecular analysis, especially in inconclusive cases, has enhanced the diagnostic power of the EUS-FNA technique [9]–[12].

Mutant *KRAS* has been reported in >90% of cases of pancreatic ductal adenocarcinoma [13] and in 30 to 45% of cases of intraductal papillary mucinous neoplasm (IPMN), a pre-malignant distinct pathological entity which is thought to be a precursor of PDAC [14]–[17]. *KRAS* mutations were not detected in acinar carcinomas of the pancreas, in pancreatic neuroendocrine tumors (pNET) or in solid pseudopapillary tumors (SPPT) [18]–[20].

*KRAS* mutations represent an early genetic event in PDAC pathogenesis and, as regards solid lesions, it is considered a tumor marker for pancreatic adenocarcinoma [21]–[23]. The detection of *KRAS* mutations in a pancreactic lesion sample is useful to confirm the preoperative diagnosis or to suggest the presence of malignancy in those cases where EUS-FNA cytology is inconclusive [11], [22], [24], [25]. Moreover it has been observed that *KRAS* point mutations could also occur in chronic pancreatitis and are associated with evolution towards pancreatic cancer [26], [27]. Several techniques could be used for *KRAS* mutation analysis, including Single-Strand Conformation Polymorphism (SSCP) [9], Restriction Fragment Length Polymorphism (RFLP) assays [28], [29], Enriched-PCR and enzyme Linked Mini-sequence Assay (ELMA-PCR) [30], clamping Peptide Nucleic Acids PCR (PNA-PCR) [31], Allele Specific Locked Nucleic Acid PCR (ASLNAqPCR) [32] and Sanger sequencing [15], [28]. Considering that cytological material obtained from EUS-FNA is often composed of heterogeneous cell populations, it is crucial to make use of accurate and high analytical sensitive molecular tests to detect even a small proportion of mutated cells in a background of wild-type ones [33].

In this work we analyzed the *KRAS* gene mutational status in 60 consecutive cases of pancreatic lesions starting from material directly collected with EUS-FNA and using three different molecular techniques. We compared Sanger sequencing (considered the gold standard technique for DNA sequence analysis) with two highly analytical sensitive and semi-quantitative techniques: ASLNAqPCR [32] and 454 Next Generation Sequencing (454 GS-Junior platform, Roche). The aim of the present study was to evaluate if a highly analytical sensitive technique could provide more accurate results (meaning fewer false negative and fewer false positive results) in the routine analysis of *KRAS* in pancreatic lesions. Moreover, considering that usually in pancreatic specimens only mutations in *KRAS* exon 2 are investigated [15], [20], [28], [34], we tested if it could be useful to analyze also *KRAS* exon 3 mutations. Finally, taking into consideration that evaluation of cellular composition is not possible from EUS-FNA material directly collected into a tube (“direct” EUS-FNA), we re-tested *KRAS* starting from cytologic smears and compared the two results, one obtained by targeting selected cells with cytologic atypia and the other directly obtained from EUS-FNA specimens.

## Materials and Methods

### Selection of Cases

Sixty samples of EUS-FNA obtained from pancreatic lesions were analyzed. According to ecoendoscopy they were classified as solid (31 cases) or cystic (29 lesions) lesions of the pancreas. Patients were 23 male and 37 female, ages ranging from 17 to 84 (mean 66 yrs).

EUS-FNA was performed using a linear echoendoscope (Fujinon, Inc., Saitama, Japan), the aspirated material was smeared on microscope slides for on-site examination or immediately fixed in 95% ethanol for Papanicolaou staining; the remaining material was partly placed in a tube containing 4% formaldehyde solution for cell block preparation and partly in a tube containing 100% ethanol for *KRAS* analysis (“direct” EUS-FNA material). Cases were diagnosed on preoperative evaluation according to standard criteria as unsatisfactory (C1), negative for malignancy (C2), atypical cells present (C3), suspicious for malignancy (C4) or positive for malignancy (C5) [35]. Regarding the unsatisfactory samples, we decided to distinguish them as C1c if the EUS-FNA was performed on a cystic lesion or C1s if EUS-FNA was performed on solid lesion. We considered C1s, C3 and C4 as inconclusive diagnoses.

Since *KRAS* mutational analysis is part of the routine diagnostic workup of patients with pancreatic lesions the need for ethic committee’s approval was not necessary for this study, in accordance with medical ethical guidelines of the Azienda Unità Sanitaria Locale di Bologna. Accordingly to these guidelines, a comprehensive written informed consent was signed for the procedure (endoscopic ultrasound fine needle aspiration) that produced the tissue samples. All information regarding the human material was managed using anonymous numerical codes. All samples were handled in compliance with the Helsinki declaration (http://www.wma.net/en/30publications/10policies/b3/).

### DNA Extraction and *KRAS* Analysis

DNA from direct EUS-FNA material or cytological smears was extracted using MasterPure DNA Purification Kit (Epincentre, Madison WI, USA) according to manufacturer’s instruction. DNA from cytoblocks was extracted using High Pure PCR Template Preparation Kit (Roche Diagnostic, Manheim, Germany). Cytological smears were considered evaluable for analysis if at least a hundred of neoplastic cells were present in the slide. The smears were scanned as virtual slides for archiving (ScanScope CS2 Digital Slide Scanner, Aperio, CA, USA) prior to dissecting.

*KRAS* mutational analysis was performed using three different techniques: Sanger sequencing, Allele Specific Locked Nucleic Acid PCR (ASLNAqPCR) and 454 Next Generation Sequencing (454-NGS).

#### Sanger sequencing

DNA was amplified using previously described primers [32], purified and sequenced for *KRAS* exon 2 and exon 3, according manufacturer’s protocol. Sequencing was carried out according to standard procedures using a CEQ2000 XL automatic DNA sequencer (Beckman Coulter, Inc., Fullerton, CA, U.S.A). Strands were analyzed using forward and reverse primers.

#### Allele specific locked nucleic acid PCR

Mutations in exon 2 were analyzed using ASLNAqPCR optimized for the 7 most common *KRAS* mutations (G12A, G12C, G12D, G12R, G12S, G12V and G13D) as previously described [32]. The percentage of mutated alleles was calculating according to the following formula [33]:
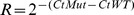
where R is the “Ratio”, Ct refers to the threshold cycle and Mut and WT refer to mutated and wild-type alleles, respectively. The analytical sensitivity of ASLNAqPCR is below 1%, as previously reported [32].

#### 454 Next-generation sequencing

Sequence analysis of *KRAS* exon 2 and exon 3 was performed with the 454 GS-Junior Next Generation Sequencer platform (Roche Diagnostics, Mannheim, Germany), according to manufacturers’ instructions. Each target sequence was read at least 300 times (“reads”). *KRAS* primers were specific for exon 2 (Fw 5′-GGCCTGCTGAAAATGACTGAA-3′; Rv 5′-TGTATCAAAGAATGGTCCTGCAC-3′) and exon 3 (5′-TCTTGGATATTCTCGACACAGCA-3′; 5′-TGCATGGCATTAGCAAAGAC-3′). A sample was considered to be mutated for *KRAS* only if mutation was present in at least 1% of the consensual reads and in at least 10 of the total reads, according to the 454-NGS analytical sensitivity previously reported [36] and also as determined by serial dilution of *KRAS* mutated cell lines (OCUT-1) (data not shown).

### Follow Up and Final Diagnosis

To determine the performance of the three different techniques, sequencing data were compared with the histological diagnosis for patients that underwent surgery. For patients that were not operated on, sequencing data were compared with a final endpoint based on a combination of clinicopathologic features and follow-up information.

According to final end-point, we distinguished three different categories of lesions: i) benign lesions, ii) adenocarcinomatous lesions (including precursor lesions of adenocarcinoma); iii) not-adenocarcinomatous lesions.

We considered non-neoplastic cysts, pseudocysts and pancreatitis as benign lesions; the inoperable neoplasias with poor progression, PDAC and intraductal papillary mucinous neoplasms (IPMN, both Branch Duct - BD-IPMN - and Main Duct - MD-IPMN) were considered as adenocarcinomatous/preneoplastic lesions; neuroendocrine (pNET) and solid pseudopapillary tumors (SPPT) were considered as not-adenocarcinomatous lesions.

### Statistical Measures of Performance

We considered a result as true positive (TP), false positive (FP), true negative (TN) or false negative (FN) as follows. TP were cases when showed a mutation in *KRAS* and which were PDAC, inoperable neoplasias or IPMN according to final end-point. FP were cases in which a mutation was found but with a “benign” endpoint or else diagnosed as SPPT or pNET. TN were cases that resulted wild-type and with a “benign” endpoint or with an endpoint of neuroendocrine or pseudopapillary neoplasia. FN were cases with a wild-type *KRAS* but were PDAC/inoperable neoplasias or IPMN at the final end-point.

Test clinical sensitivity (SEN), specificity (SPEC), negative predictive value (NPV), positive predictive value (PPV), accuracy (ACC) and false discovery rate (FDR) were calculated as previously described [32]. Comparisons between clinical sensitivities were performed according to recommendations previously described [37]. Results with a p-value of <0.05 were considered to be statistically significant.

## Results

Cytologic results and features, final end-point and *KRAS* molecular analysis are summarized in Tables 1 and 2.

**Table 1:** Percentage of mutated *KRAS* samples according to preoperative cytology evaluation.

Cytology Diagnosis (number of cases)	*KRAS* mutated samples					
	454 NGS (%)	End-point of mutated samples	ASLNA (%)	End-point of mutated samples	Sanger sequencing (%)	End-point of mutated samples
C1 (20)	8 (40.0)	6 IPMN (4 BD, 2 MD), 2 NA	4 (20.0)	2 IPMN (BD), 2 NA	4 (20.0)	3 IPMN (2 BD, 1 MD), 1 NA
C1c (17)	8 (47.1)		4 (23.5)		4 (23.5)	
C1s (4)	0 (0)		0 (0)		0 (0)	
C2 (4)	0 (0)		0 (0)		0 (0)	
C3 (2)	1 (50.0)	Mal. Inop. Neoplasia	1 (50.0)	1 Mal. Inop. Neoplasia	1 (50.0)	1 Mal. Inop. Neoplasia
C4 (9)	7 (77.8)	5 PDAC, 1 IPMN (BD), 1Mal. Inop. Neoplasia	6 (66.7)	4 PDAC, 1 Mal. Inop.Neoplasia, 1 IPMN (BD)	5 (55.6)	3 PDAC, 1 Mal. Inop. Neoplasia, 1 IPMN (BD)
C5 (20)	11 (55.0)	9 PDAC, 2 Mal. Inop. Neoplasia	10 (50.0)	8 PDAC, 2 Mal. Inop.Neoplasia	6 (30.0)	5 PDAC, 1 Mal. Inop. Neoplasia
C5 PDAC (13)	11 (84.6)		10 (76.9)		6 (46.2)	
C5 Not PDAC. (7)	0 (0)		0 (0)		0 (0)	
NA (5)	4 (80.0)	3 IPMN (2 BD, 1 MD), 1 NA	3 (60.0)	2 IPMN (1 BD, 1 MD), 1 NA	1 (20.0)	1 IPMN (BD)
**TOTAL (60)**	**31 (51.7)**		**24 (40.0)**		**17 (28.3)**	

ASLNAqPCR, Allele Specific Locked Nucleic Acid qPCR; NGS, Next Generation Sequencing; PDAC, Pancreatic Ductal AdenoCarcinoma; Not PDAC, malignant neoplasia but not Pancreatic Ductal Adenocarcinoma; NA, cytology not available.

**Table 2:** Percentage of mutated *KRAS* samples according to different techniques per final end-point.

	Number of *KRAS* mutated samples using:		
Final End-Point	454 NGS (%)	ASLNAqPCR (%)	Sanger (%)
**Adenocarcinomatous and pre-neoplastic lesions (n = 38)**	28 (73.7)	21 (55.3)	16 (42.1)
*** PDAC (n = 20)***	*14 (70)*	*12 (60)*	*8 (40)*
*** IPMN (n = 12)***	*10 (83.3)*	*5 (41.7)*	*5 (41.7)*
*** Inop. Neoplasia (n = 6)***	*4 (66.7)*	*4 (66.7)*	*3 (50)*
**Not-adenocarcinomatous lesions (n = 7)**	0 (0)	0 (0)	0 (0)
*** pNET (n = 5)***	0 (0)	0 (0)	0 (0)
*** SPPT (n = 2)***	0 (0)	0 (0)	0 (0)
**Benign Lesions (n = 12)**	0 (0)	0 (0)	0 (0)
**NA (n = 3)**	3 (100)	3 (100)	1 (33.3)

PDAC, Pancreatic Ductal AdenoCarcinoma; IPMN, Intraducatal Pancreatc Mucinous Neoplasia; Inop. Neoplasia, Malignant inoperable neoplasia; pNET, pancreatic NeuroEndocrine Tumor; SPPT, Solid PseudoPapillary Tumor; NA, end-point not available.

### Cytologic Evaluation (Table 1)

Material for cytologic evaluation was available for 55 cases. According to preoperative cytology diagnosis, specimens were classified as follows: unsatisfactory (C1, 20 cases), negative for malignancy (C2, 4 cases), atypical cells present (C3, 2 cases), suspicious for malignancy (C4, 9 cases), positive for malignancy (C5, 20 cases). Among the latter, 13 were PDAC, 5 were pNET and 2 were SPPT. The samples diagnosed as C1 were considered C1c (17 cases) if the EUS-FNA material was obtained from a cystic lesion or C1s (3 cases) if it was evaluated from a solid lesion. A virtual slide of one of the specimens analyzed is available at the following address: http://vetrinodigitale.ausl.bo.it/spectrum_Login.php (username and password are available upon request).

### *KRAS* Sequencing in Direct EUS-FNA Material (Tables 1–2)

#### Sanger sequencing

**Figure 1:**
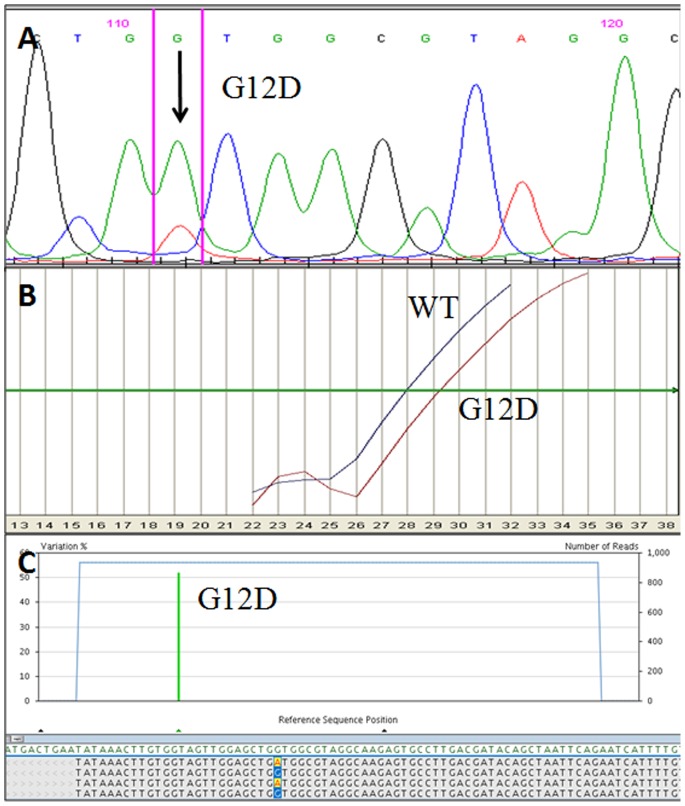
Example of molecular results in a *KRAS* exon 2 mutated sample. A) Electropherogram obtained using Sanger sequencing. The *KRAS* G12D mutation is identified by the smaller peak pointed by the arrow. B) Using ASLNAqPCR results the *KRAS* G12D mutation is identified by the right curve (G12D). The left curve indicates the wild-type allele (WT). C) Profile obtained using 454-NGS, the *KRAS* G12D mutation is identified by the vertical green bar. The percentage of mutated alleles is indicated on the left y axis while the total number of reads on the right one.

**Figure 2:**
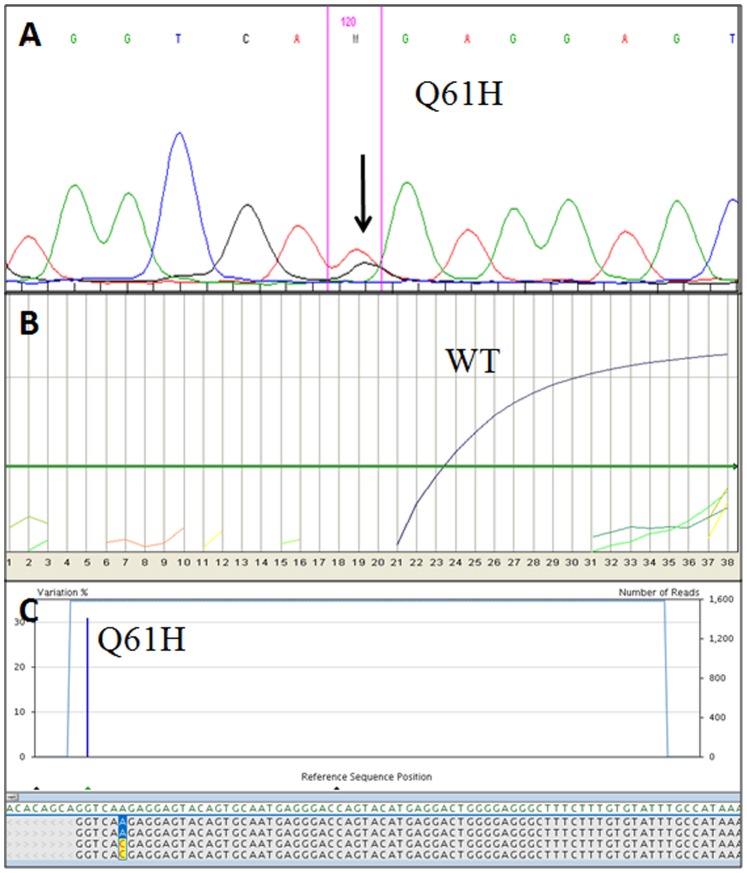
Example of molecular results in a *KRAS* exon 3 mutated sample (case #37, Table 4). A) Electropherogram obtained using Sanger sequencing. The *KRAS* Q61H mutation is identified by the smaller pick pointed by the arrow. B) No *KRAS* mutation was detected using ASLNAqPCR results: only the curve corresponding to the wild-type allele is visible (WT). C) Profile obtained using 454-NGS, the *KRAS* Q61H mutation is identified by the vertical blue bar. The percentage of mutated alleles is indicated on the left y axis while the total number of reads on the right one.

Using Sanger sequencing, 17 of 60 samples (28.3%) showed a mutation in *KRAS* exon 2 (15 of 60 cases, 25%) or in the exon 3 (2 of 60 cases, 3.3%) (Figures 1–2).

In particular these latter cases were a C1 cystic lesion (mutated for Q61H) and a C4 suspected for malignancy (mutated for Q61L) which were respectively a BD-IPMN with low-grade dysplasia and a PDAC upon follow-up. In two cases, double mutations in KRAS exon 2 were observed (see “Multiple *KRAS* mutations” paragraph).

As summarized in Table 1, *KRAS* mutations were found in the 20.0% of inadequate samples (C1), in one of the two cases (50.0%) with atypical cells (C3), in the 55.6% of the cases suspect for malignant neoplasia (C4) and in the 30.0% of samples diagnosed as C5. All C2 cases showed no mutations in the *KRAS* gene and they were benign cysts (3 cases) or pancreatitis (one case) on follow-up. One of five cases with no available material for cytological evaluation was mutated for *KRAS* and it was a BD-IPMN on follow-up.

Considering the final endpoint, using Sanger sequencing we detected a *KRAS* mutation in 42.1% of adenocarcinomatous and pre-neoplastic lesions (in 40% of PDAC, 41.7% of IPMNs and in the 50% of inoperable neoplasms), while no *KRAS* mutations were observed in not adenocarcinomatous or in benign lesions (Table 2).

#### ASLNAqPCR

Using ASLNAqPCR analysis, 24 of 60 samples (40.0%) showed a mutation in *KRAS* exon 2 (Figure 1, Table 1). All mutations in *KRAS* exon 2 detected by Sanger sequencing were also detected by ALSNAqPCR (Figures 1A–1B). In five cases, double mutations in *KRAS* exon 2 were observed (see “Multiple *KRAS* mutations” paragraph).

No *KRAS* exon 3 (codon 61) mutations were detected by ASLNAqPCR because this technique is designed only for the 7 most common mutations in *KRAS* codon 12–13 [32].

As summarized in Table 1, *KRAS* mutations were found in the 20.0% of inadequate samples (C1), in one of the two cases (50.0%) with atypical cells (C3), in the 66.7% of the cases suspect for malignant neoplasia (C4) and in ten cases diagnosed as malignant neoplasia (C5). All the C2 cases showed no *KRAS* mutations. Three cases with no available material for cytological evaluation were mutated for *KRAS.* Upon follow-up two were IPMN, one BD-IPMN and one MD-IPMN with moderate dysplasia. No further information was available in the third case).

Considering the final endpoint, using ASLNAqPCR we detected a *KRAS* mutation in the 56.8% of adenocarcinomatous and pre-neoplastic lesions (in the 60% of PDAC, 41.7% of IPMNs and in the 66.7% of inoperable neoplasias. No *KRAS* mutations were observed in not adenocarcinomatous or in benign lesions (Table 2).

#### 454 Next-generation sequencing

Using 454-NGS, 31 of 60 samples (51.7%) showed a mutation in *KRAS* exon 2 (24 of 60, 40%) and/or in *KRAS* exon 3 (10 of 60, 16.7%) (Figures 1–2). Raw data are available upon request.

All mutations in *KRAS* exon 2 detected by Sanger sequencing and ALSNAqPCR were also detected using 454-NGS (Figures 1 A–C).

In 7 cases (four C1c, one C4, one C5; in one case no material was available for cytologic evaluation) only a *KRAS* exon 3 (codon 61) substitution was found, while in 3 cases (two C4 and one C5) a *KRAS* exon 3 substitution was found in association with a mutation in *KRAS* exon 2 (see “*Multiple KRAS mutations*” paragraph). All mutations in *KRAS* exon 3 detected by Sanger sequencing were also detected using 454-NGS.

*KRAS* mutations were found the 40.0% of inadequate samples (C1), in one of the two cases (50.0%) with atypical cells (C3), in the 77.8% of cases suspect for malignant neoplasia (C4) and in eleven cases diagnosed as malignant neoplasia (C5) (Table 1). All the C2 cases showed no mutations in the *KRAS* gene. Four cases with no available material for cytologic evaluation were mutated for *KRAS* and upon follow-up three were IPMN (two BD-IPMN and one MD-IPMN with moderate dysplasia). No further information was available in the third case (Table 1).

Considering the final endpoint, using 454-NGS we detected a *KRAS* mutation in the 75.7% of adenocarcinomatous and pre-neoplastic lesions (in the 70% of PDAC cases, in the 83.3% of IPMNs and in the 66.7% of inoperable neoplasias). No *KRAS* mutations were observed in not adenocarcinomatous or in benign lesions (Table 2).

### Multiple *KRAS* Mutations

Using Sanger sequencing double mutations of the *KRAS* gene exon 2 were observed in two cases (one C4 and one C5) that were PDAC upon follow-up (Table 3). ASLNAqPCR allowed to detect multiple mutations in 5 of 24 mutated cases (one C4 and two C5; in two cases cytologic evaluation was not available). These five cases were one IPMN and five PDAC upon follow-up (Table 3). Finally, next generation sequencing analysis allowed to observe multiple mutation of *KRAS* gene in 7 of 31 mutated cases (two C4 and three C5; in two cases no cytology material was available). In 3 of these 31 mutated cases, mutations in *KRAS* exon 2 and in *KRAS* exon 3 were observed. Upon follow-up, these seven cases were one IPMN and six PDAC (Table 3).

**Table 3:** Multiple *KRAS* mutations according to different techniques.

Technique (#sample)	Mutations	Final End-Point
**Sanger sequencing**		
** #4**	G12D/G12V	PDAC
** #53**	G12D/G12V	PDAC
**ASLNAqPCR**		
** #4**	G12D/G12V	PDAC
** #5**	G12D/G12V	PDAC
** #22**	G12D/G12V	IPMN
** #26**	G12C/G12V	NA
** #53**	G12C/G12D/G12R/G12V	PDAC
**454 NGS**		
** #4**	G12D/G12V	PDAC
** #5**	G12D/G12V	PDAC
** #6**	G12V/Q61H	PDAC
** #22**	G12D/G12V	IPMN
** #26**	G12C/G12V	NA
** #53**	G12C/G12D/G12R/G12V/Q61H	PDAC
** #54**	G12D/Q61H	PDAC

PDAC, Pancreatic Ductal AdenoCarcinoma; IPMN, Intraducatal Pancreatc Mucinous Neoplasia; NA, end-point not available.

### Discrepant *KRAS* Results between Sanger Sequencing, ASLNAqPCR and 454-NGS

Results of discrepant cases are summarized in Table 4.

**Table 4:** Discrepant results obtained with the three techniques.

Cytological (preoperative) Diagnosis (# of consecutive case)	*KRAS* mutational status			Final End-Point
	454 NGS (% ofmutated reads)	ASLNAqPCR (Ratio)	Sanger sequencing	
**C1**				
#31	Q61H (4.3)	WT	WT	BD-IPMN
#37	Q61H (31.0)	WT	Q61H	MD-IPMN
#42	G12V (2.7)	G12V (0.02)	WT	NA
#44	Q61L (3.0)	WT	WT	BD-IPMN
#46	Q61R (1.1)	WT	WT	MD-IPMN
**C4**				
#52	Q61L (22.0)	WT	Q61L	PDAC
#57	G12D (6.5)	G12D (0.03)	WT	PDAC
#60	G12R (12.0)	G12R (0.06)	WT	PDAC
**C5**				
#8	G12D (19.3)	G12D (0.01)	WT	PDAC
#9	Q61H (15)	WT	WT	PDAC
#10	G12V (1.5)	G12V (0.08)	WT	PDAC
#11	G12V (1.0)	G12V (0.02)	WT	PDAC
#14	G12D (3.0)	G12D (0.02)	WT	Malignant Inop. Neop^1^
**NA**				
#21	G12V (3.7)	G12V (0.02)	WT	MD-IPMN
#25	Q61H (1.4)	WT	WT	BD-IPMN
#26	G12C&G12V (2.6&2.0)	G12C&G12V (0.02&0.01)	WT	NA

1 Malignant Inop. Neop, Malignant Inoperable Neoplasia, patient did not undergo surgery, FU determined according to clinical data. FU, Follow-Up; NGS, Next Generation Sequencing; ASLNAqPCR, Allele Specific Locked Nucleic Acid qPCR; WT, Wild-Type; PDAC, Pancreatic Ductal AdenoCarcinoma; IPMN, Intraductal papillary mucinous neoplasm; BD, Branch Duct; MD, Main Duct; NA, follow-up not available.

In 16 cases discordant results in *KRAS* mutational status were obtained using at least one of the three different techniques (Table 4). Upon cytologic evaluation, 5 of 16 cases were diagnosed as malignant (C5) and 3 as suspect for malignancy (C4). In 5 cases the samples were cyst content material considerate inadequate for cytologic diagnosis (C1c). In three cases no material was available for cytologic examination.

#### C1 cases

In one sample a mutation in *KRAS* exon 2 gene was detected using 454-NGS and ASLNAqPCR, but not with Sanger sequencing (#42, Table 4). No follow up was available for this case. In one sample a mutation in *KRAS* exon 3 was detected both with 454-NGS and Sanger sequencing (#37, Table 4); it was a MD-IPMN (with low-grade dysplasia) according to histological evaluation. In the three remaining cases (#31, #44 and #46, Table 4) a mutation in *KRAS* exon 3 was detected only using 454-NGS and all three cases were IPMN (two BD-IPMN and one MD-IPMN) after post-operative histologic evaluation (Figures 2 A–C).

#### C4 cases

**Figure 3:**
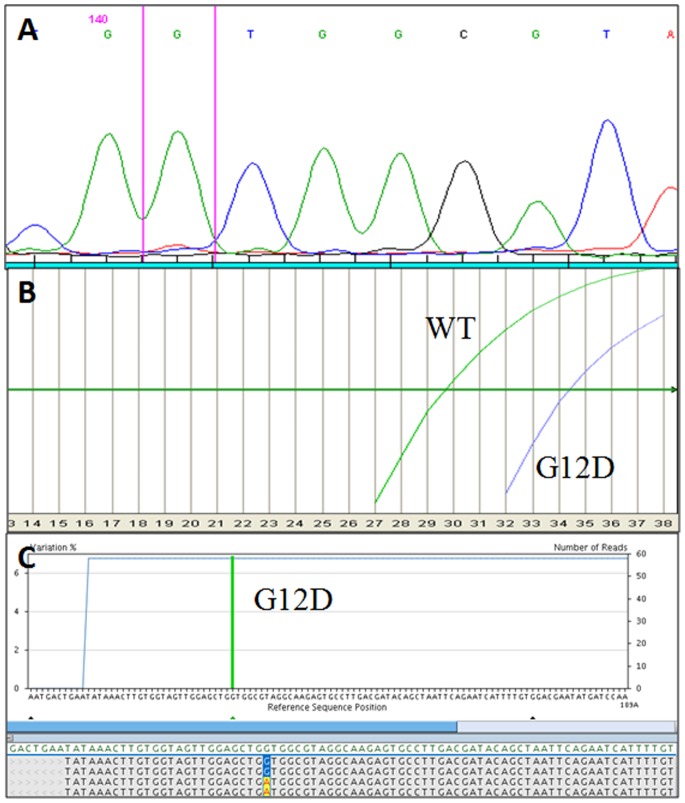
Example of molecular results in a *KRAS* exon 2 mutated sample (case #57, Table 4) with discordant results between the three techniques. A) Electropherogram obtained using Sanger sequencing. The mutation is not identified. B) Using ASLNAqPCR the *KRAS* G12D mutation is identified by the right curve (G12D). The left curve indicates the wild-type allele (WT). The ratio between the two curves corresponds to ∼6% of mutated alleles. C) Profile obtained using 454-NGS, the *KRAS* G12D mutation is identified by the vertical green bar. The percentage of mutated alleles is indicated on the left y axis while the total number of reads on the right one.

In two C4 samples (#57 and #60, Table 4) a mutation in *KRAS* exon 2 gene was detected using 454-NGS and ASLNAqPCR, but not with Sanger sequencing (Figures 3 A–C). All cases were pancreatic ductal adenocarcinoma (PDAC) after post-operative histologic evaluation. In one case (#52, Table 4) a mutation in *KRAS* exon 3 was detected both with 454-NGS and Sanger sequencing but not using ASLNAqPCR. It was a PDAC according to histological evaluation.

#### C5 cases

In four C5 cases (#8, #10, #11 and #14, Table 4) a mutation in *KRAS* exon 2 gene was detected using 454-NGS and ASLNAqPCR, but not with Sanger sequencing. All cases were malignant carcinoma upon follow-up: three were diagnosed as pancreatic ductal adenocarcinoma (PDAC) after post-operative histologic evaluation, one case did not undergo surgery and was considered a malignant primary pancreatic neoplasm, based on clinical findings. In one case (#9, Table 4) a mutation in *KRAS* exon 3 was detected only with 454-NGS and was found to be a PDAC after post-operative histologic evaluation.

#### Cases with cytologic evaluation not available

All three samples with no cytologic evaluation and discrepant *KRAS* results were from cystic lesions. In two cases (#21 and #26, Table 4) at least one mutation in *KRAS* exon 2 gene was detected using 454-NGS and ASLNAqPCR, but not using Sanger sequencing. According to follow-up, one case (#21, Table 4) was a MD-IPMN (with moderate dysplasia). In the other no follow-up information was available. In the third case (#25, Table 4) a mutation in *KRAS* exon 3 was detected only with 454-NGS and the lesion was diagnosed as BD-IPMN after post-operative histologic evaluation.

According to the percentage of mutated reads (454-NGS) and ratio values (ASLNAqPCR) all the mutations in *KRAS* exon 2 detected using 454-NGS and ASLNAqPCR but not using Sanger sequencing were below the Sanger analytical sensitivity threshold (∼20% of mutated alleles, corresponding to ∼40% of mutated cells, considering the mutation heterozygous) (Table 4). The five mutations detected in *KRAS* exon 3 only by 454-NGS were all below the Sanger sequencing analytical sensitivity threshold (Table 4). On the contrary, the two *KRAS* exon 3 mutations detected both using 454-NGS and Sanger sequencing were observed in more than 20% of the total reads (Table 4).

### *KRAS* Mutational Status in Cytologic Smears

Considering that it was not possible to determine the proportion or the presence of neoplastic cells in EUS-FNA material directly collected into a tube, we decided to re-analyze the samples not mutated for *KRAS* starting from cytological smears or cyto-blocks. The slides suitable for the manual dissection of neoplastic cells were selected by a pathologist and the representative area was marked. Residual material for molecular analysis was available for 24 of 29 patients that were wild-type for *KRAS* according all three techniques. On cytologicic evaluation, these 24 samples were diagnosed as: C1 in ten cases, C2 in 3 cases, C3 in one case, C4 in two cases and C5 in 8 cases (Table 5). In the 13 cases evaluated as unsatisfactory (C1c or C1s) or negative for malignancy (C2) the re-analysis was not performed. Due to the higher clinical sensitivity of NGS (see “Statistical measures of performance” paragraph) the analysis was repeated only using 454-NGS.

**Table 5:** Molecular results of *KRAS* analysis in material obtained from cytological smears.

Number of consecutive analyzed Cases	Pre-operative Diagnosis	*KRAS* status onFNA material	*KRAS* status on cytological smear/cytoblock (% of mutated reads)	Final End-Point
#1	C5	WT	G12C (1.3)	PDAC
#3	C5	WT	WT	Malignant Inop. Neop.^1^
#7	C5	WT	WT	pNET
#15	C5	WT	WT	pNET
#16	C5	WT	WT	pNET
#17	C5	WT	WT	pNET
#50	C5	WT	WT	SPPT
#51	C5	WT	WT	SPPT
#19	C4	WT	WT	PDAC
#55	C4	WT	WT	Malignant Inop. Neop.^1^
#43^2^	C3	WT	G12V (1.7)	PDAC

FNA, Fine Needle Aspiration; FU, Follow-Up; PDAC, Pancreatic Ductal AdenoCarcinoma; pNET, pancreatic NeuroEndocrine Tumor; SPPT, Solid PseudoPapillary Tumor.

1 Malignant Inop. Neop, Malignant Inoperable Neoplasia, no histological evaluation was possible.

2 A digital slide of this sample is available at the following address http://vetrinodigitale.ausl.bo.it/spectrum_Login.php.

Two further mutations in the *KRAS* gene were observed in material obtained from cytologic smears. A *KRAS* G12V mutation was observed in a sample with “atypical cells” (C3) upon cytologic evaluation (#43 Table 5). This case was a PDAC according to post-operative histologic evaluation. A *KRAS* G12C mutation was detected in a sample with a “malignant” cytologic diagnosis (C5) (#1 Table 5) that was an adenocarcinoma after post-operative histologic evaluation. The remaining cases were wild-type for *KRAS*, even after the analysis was repeated on material dissected from the cytology specimen. According to follow-up these cases were PDAC (one case), pNET (4 cases), SPPT (2 cases). Two were inoperable malignant neoplasms (Table 5).

### Statistical Measures of Performance

As shown in Table 6, when the analysis of *KRAS* (exon 2 and exon 3) was performed on direct FNA the 454-NGS, ASLNAqPCR and Sanger sequencing had 100% specificity. If only *KRAS* exon 2 was analyzed, the clinical sensitivity of 454-NGS (52.78%) was higher (p<0.001) than Sanger sequencing (44.19%) and comparable with that of ASLNAqPCR (52.78%). Clinical sensitivity (73.68%, p<0.001), negative predicted value (65.52%) and accuracy (82.46%) of 454-NGS were higher if *KRAS* exon 3 was also analyzed (Table 6).

**Table 6:** Statistical performance of *KRAS* molecular analysis using the three different techniques.

	*KRAS* Ex 2			*KRAS* Ex 2 and Ex 3		
*Performance*	454 NGS	ASLNA	Sanger	454 NGS	ASLNA	Sanger
*SPEC (%)*	**100.00**	**100.00**	**100.00**	**100.00**	**100.00**	**100.00**
*SENSIT (%)*	52.78	52.78	44.19	**73.68**	52.78	42.11
*PPV (%)*	**100.00**	**100.00**	**100.00**	**100.00**	**100.00**	**100.00**
*NPV (%)*	55.26	55.26	36.84	**65.52**	55.26	46.34
*ACC (%)*	70.18	70.18	57.89	**82.46**	70.18	70.18
*FDR (%)*	0.00	0.00	0.00	0.00	0.00	0.00

Ex, Exon; NGS, Next Generation Sequencing; ASLNA, Allele Specific Locked Nucleic Acid qPCR; Sanger, Sanger sequencing; SPEC, Specificity; SENS, Clinical Sensitivity; PPV, Positive Predictive Value; NPV; Negative Predictive Value; ACC; Accuracy; FDR, False Discovery Rate. In bold the higher value per each parameter.

The repeated analysis using 454-NGS of *KRAS* wild-type samples starting from DNA obtained from cytologic specimens led to increases in clinical sensitivity (78.95%, p<0.05), negative predictive value (70.37%) and accuracy (85.96%) (Table 7).

**Table 7:** Statistical performance of *KRAS* molecular analysis using 454-NGS starting only from FNA material or adding the results obtained in DNA extracted from cytologic smears (in bold).

*454 NGS performances*	*KRAS* analysis only on direct EUS-FNA sample	*KRAS* analysis performed also on cells scraped from the cytologic smears or cytoblocks
*SPEC (%)*	100.00	**100.00**
*SENS (%)*	73.68	**78.95**
*PPV (%)*	100.00	**100.00**
*NPV (%)*	65.52	**70.37**
*ACC (%)*	82.46	**85.96**
*FDR (%)*	0.00	**0.00**

SPEC, Specificity; SENS, Clinical Sensitivity; PPV, Positive Predictive Value; NPV; Negative Predictive Value; ACC; Accuracy; FDR, False Discovery Rate.

## Discussion

Detection of *KRAS* mutations can be used to improve the pre-operative diagnosis of pancreatic EUS-FNA samples. For this reason it is of crucial importance to perform the analysis with highly specific and analytical sensitive techniques. Sanger sequencing has been widely used for *KRAS* analysis, but the low analytical sensitivity (∼20% of mutated alleles) of the method can lead to false negative results [32] if compared with highly sensitive methods. Mutation specific assays, as ASLNAqPCR, notably improve the analytical sensitivity of *KRAS* molecular analysis, allowing to recognize 1% of mutated alleles [32]. However, these assays are designed only for particular “hot-spot” *KRAS* mutations (e.g. in *KRAS* exon 2). They can therefore underestimate the number of mutated samples, an example being those specimens with substitutions in *KRAS* exon 3 that are not usually targeted by mutation specific assays. Next generation sequencing merges the high analytical sensitivity of mutation specific assays with the broader capabilities of direct sequencing methods like Sanger, that not being mutation specific also allows the detection of unusual or unexpected mutations. Our data demonstrate that 454-NGS has a higher clinical sensitivity (73.68%) than ASLNAqPCR (52.78%, p<0.001) and Sanger sequencing (42.11%, p<0.001) in *KRAS* mutation detection of pancreatic lesions starting from EUS-FNA material. Mutations in specimens with a low proportion of mutated alleles (<20%, corresponding to <40% of cells considering the mutation heterozygous) were detected only using 454-NGS and ASLNAqPCR (if the mutation was present in exon 2) or only using 454-NGS (if the mutation was in *KRAS* exon 3). These results are fully compatible with the fact that that Sanger sequencing can detect a mutation only if it is present in more than 40% of the cells.

Our analysis of *KRAS* using highly analytical sensitive techniques such as ASLNAqPCR or 454-NGS not only increases the clinical sensitivity of the test but also maintains a very high level of specificity (100%).

Even if *KRAS* exon 2 mutations were still the majority (40% of all analyzed cases using NGS), mutations of *KRAS* exon 3 were also observed many samples (16.7% using NGS). Using Sanger sequencing our percentage of *KRAS* exon 3 mutated cases was about 3%, similar to that generally reported in the literature [38]). In three cases the *KRAS* exon 3 mutation would not have affected the molecular diagnosis, considering that the mutation was found in association with another *KRAS* mutation in exon 2. However, in seven cases the mutation in *KRAS* exon 3 was the only alteration found. All these seven cases would have been considered wild-type for *KRAS* although, on follow-up, five were IPMN and two were PDAC. For this reason, the detection of additional mutation in *KRAS* exon 3 provides very useful diagnostic information. Moreover, when both *KRAS* exon 2 and exon 3 were analyzed, the clinical sensitivity of 454-NGS was higher (73.68%) than when only *KRAS* exon 2 was investigated (52.78%).

IPMN is a spectrum of neoplasms in the pancreatic duct epithelium characterized by cystic dilation of the main pancreatic ducts and/or of their branches. IPMN is considered to be a precursor of PDAC. It has been proposed that the process of IPMN follows an adenoma - carcinoma sequence and that the time of progression to malignancy is about 5 years [39]. Since the process is slow, a correct diagnosis of IPMN provides the opportunity to cure the patient, before an invasive preoperative adenocarcinoma develops.

The results presented here indicate not only that IPMN are frequently mutated for *KRAS* (83.3% in our series, consistent with other studies that analyze only *KRAS* exon 2 in IPMN [14], [15], [40]), but also that they are commonly mutated in *KRAS* exon 3 (41.7%).

Analysis of *KRAS* starting from direct EUS-FNA material allows to obtain good quality DNA for molecular analysis that is therefore available at the same time of the cytologic evaluation. In samples with unsatisfactory cytology (e.g. cyst content material, C1c), the analysis of *KRAS* from EUS-FNA material directly collected into a tube is the only way to evaluate the mutational status of the gene. However, it is important to note that the direct analysis of EUS-FNA does not allow for the determination of the presence or the proportion of neoplastic cells in the specimen. Since this type of analysis is “blind”, DNA could originate from a population of cells not representative of the lesion, resulting in false negative results.

For this reason, in samples directly collected in a tube with a wild-type *KRAS* result it is important to repeat the analysis after the dissection of diagnostic material from the corresponding cytology specimen (smear or cytoblock) if there are atypical cells in the cytology preparation. In fact, in our series two cases found to be wild-type for *KRAS* starting from direct EUS-FNA material resulted mutated after the analysis was repeated on cells scraped from the cytologic smears. Both cases were diagnosed as adenocarcinoma after postoperative histologic evaluation.

**Figure 4:**
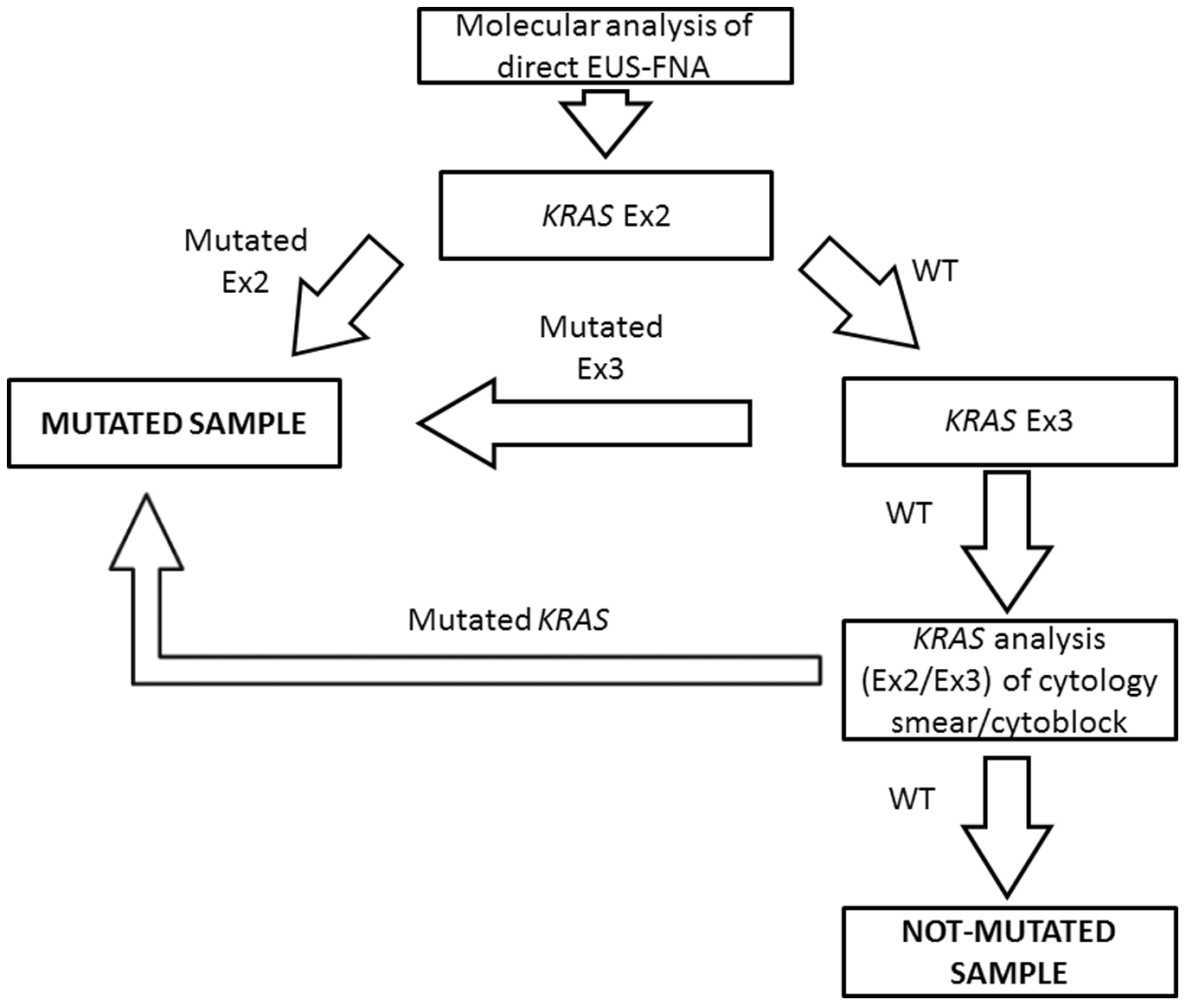
Proposed algorithm for the detection of *KRAS* mutations in EUS-FNA material from pancreatic lesions. Ex, Exon; WT, Wild-Type.

We propose an algorithm for *KRAS* analysis of pancreatic lesions (Figure 4). Next generation sequencing is more labor intensive and time-consuming that mutation specific techniques, as ASLNAqPCR. The turn-around time of 454-NGS depends on the throughput of the laboratory and batches of at least 100 amplicons (targeting the same or different exons) have to be run to lower the cost of the sequence to ∼20€ per amplicon. One hundred amplicons correspond to the analysis of 50 cases if both *KRAS* exons 2 and 3 are analyzed, for a total cost of ∼40€ per patient.

To reduce turnaround time, the *KRAS* analysis could be performed by initially studying only *KRAS* exon 2, using a highly analytical sensitive mutation-specific method that is not time-consuming (less than 2 working days, including DNA extraction). This molecular analysis is carried out in conjunction and at the same time of the cytologic evaluation. If the sample is not mutated, *KRAS* analysis should be expanded to include *KRAS* exon 3, using a highly analytical sensitive direct sequencing technique such as pyrosequencing or Next Generation sequencing. If the sample is still not mutated for *KRAS* and the cytologic smear shows atypical, suspicious or malignant cells, *KRAS* analysis should be repeated starting from cells dissected from the cytology smear or cytoblock (Figure 4).

The algorithm we propose allowed us to reach a clinical sensitivity of ∼80% and a remarkable negative predictive value of ∼70%, if cases that were wild type for *KRAS* starting from material directly collected with EUS-FNA were re-analyzed on selected material dissected from the cytology smear or cytoblock. We want to point out that this high clinical sensitivity was achieved without any false positive result, with a 100% of specificity.

Four routine clinical test it is desirable, if not mandatory, to perform DNA analysis only after careful morphologic evaluation of the cytologic or histologic material submitted for molecular diagnosis. In fact, a negative *KRAS* result on a sample that consists of non-neoplastic cells or that is inadequate must be considered non-informative for clinical purposes. The results obtained in the present study stress the importance of morphologic evaluation of the material analyzed to detect *KRAS* mutations.

Finally, the analysis of *KRAS* with a semi-quantitative method (454-NGS or ASLNAqPCR), performed on material of well-known cellular composition, allows to clarify if mutations (single or multiple) are present in a small percentage of tumor cells (sub-clones) or in the vast majority of them as in the case of all “driver” mutations [33]. This type of evaluation is obviously not possible starting from direct EUS-FNA material.

In conclusion, our study underlines the importance of using a highly analytical sensitive technique for *KRAS* – as well as for any other molecular marker - mutation analysis to support the pathologist in the diagnosis of pancreatic lesions, as also recently shown in a meta-analysis by Fuccio et al. [42]. In this series Next Generation Sequencing has allowed us to reach a very high clinical sensitivity without getting false positive results. Highly analytical sensitive *KRAS* mutation analysis can prevent repeat biopsies and thus improve patient care while reducing costs [41], [42]. Considering the high prevalence of *KRAS* codon 6 mutation, particularly in IPMN, the analysis *KRAS* exon 3 should be performed in all pancreatic lesions.

Finally, analysis of wild-type *KRAS* samples should be repeated starting from cells dissected from selected cytology specimens (smears or cytoblocks), since direct EUS-FNA material can provide false negative results.
